# MetOrigin: Discriminating the origins of microbial metabolites for integrative analysis of the gut microbiome and metabolome

**DOI:** 10.1002/imt2.10

**Published:** 2022-03-21

**Authors:** Gang Yu, Cuifang Xu, Danni Zhang, Feng Ju, Yan Ni

**Affiliations:** ^1^ The Children's Hospital, Zhejiang University School of Medicine National Clinical Research Center for Child Health Hangzhou Zhejiang China; ^2^ Key Laboratory of Coastal Environment and Resources of Zhejiang Province, School of Engineering Westlake University Hangzhou Zhejiang China

**Keywords:** correlation analysis, gut microbiome and metabolome, metabolic pathway enrichment analysis, origins of metabolites, Sankey network

## Abstract

The interactions between the gut microbiome and metabolome play an important role in human health and diseases. Current studies mainly apply statistical correlation analysis between the gut microbiome and all the identified metabolites to explore their relationship. However, it remains challenging to identify the specific metabolic functions of microbes without in vitro culture experiments for validation. Discriminating the microbial metabolites from others (e.g., host, food, or environment) and exploring their metabolic functions and correlations with microbiome specifically may improve the efficiency and accuracy of biomarker discovery. So far, there have been no such bioinformatics tools available. Herein, we developed MetOrigin, an interactive web server that discriminates metabolites originating from the microbiome, performs the origin‐based metabolic pathway enrichment analysis, and integrates the statistical correlations and biological relationships in the database using Sankey network visualization. MetOrigin not only enables the quick identification of microbial metabolites and their metabolic functions but also facilitates the discovery of specific bacterial species that are closely associated with metabolites statistically and biologically. MetOrigin is freely available at http://metorigin.met-bioinformatics.cn/.

## Highlights

- MetOrigin provides the quick identification of microbiota‐related metabolites and their metabolic functions in metabolomics studies.
- MetOrigin integrates statistical and biological correlations using Sankey network visualization to identify specific microbes associated with metabolites of interest.
- MetOrigin is a web server of microbiome and metabolome data integration analysis, and it is freely available at http://metorigin.met-bioinformatics.cn/.

## Graphical abstract

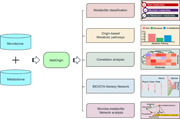

## INTRODUCTION

The fundamental role of the human metabolome and microbiome has been interrogated in many diseases including obesity, inflammatory bowel disease, cardiovascular diseases, neurodegenerative diseases, and cancers [1]. The gut microbiota contributes to host physiology by producing a diverse array of small molecule metabolites from dietary nutrients, drugs, and host‐derived components, such as short‐chain fatty acids, amino acids, bile acids, and so forth [2]. These metabolites in return play important roles in keeping the microbial homeostasis, that is, shaping the development of energy metabolism, immune system, and pathogens for the host [3, 4]. Numerous studies have identified the diagnostic and prognostic merits of microbial metabolite biomarkers that are relevant to host health [2]. Despite the complexity of these metabolites present in the host‐microbiota microenvironment, three origins can be summarized as follows: synthesized de novo by microbes, produced by microbiota from dietary components, and metabolized by host followed with microbe modifications [5]. However, with the limit of our knowledge, it is challenging for us to understand the metabolic capacity of microbes, the microbial participation in certain metabolic reactions, or even difficult to distinguish whether metabolites originate from the microbial community or host.

Many studies perform metabolomics and metagenomics analysis simultaneously and then explore their relationship between metabolites and microbiome using statistical correlation analysis methods [6]. The deep exploration of biological significance requires extensive literature searching and a series of in vivo and in vitro experiments. Therefore, bioinformatics work is urgently required in this field to incorporate valuable prior biological knowledge into an efficient statistical analysis pipeline to smooth the annotation of metabolite origins and obtain a systemic‐level understanding of microbiome and metabolome interactions. Several bioinformatics studies have taken initial steps to address this challenge, such as MIMOSA to predict the effect of an ecological community on metabolite concentrations [7] and AMON to distinguish contributions from microbiome or host to metabolite levels [8]. Based on these initial efforts to emphasize the contributions of different organisms toward metabolites, it is valuable to take further steps to discriminate the metabolic activities belonging to the host or bacterial community. Otherwise, it is difficult to understand the true metabolic functions of microbial metabolites when all the metabolites from the host and bacterial communities are pooled together, which has been unfortunately conducted frequently in many previous studies. Also, while statistical correlation analysis between microbe–metabolite pairs can guide us to explore the novel relationship, biological interpretation will stimulate us to better understand the potential metabolic activity of an organism. Thus, it is important to integrate both statistical and biological relationships together to explore the complex microbiome–metabolome network. However, it still lacks a bioinformatics pipeline in this field to validate the statistical correlations through searching the metabolic functions or activities of bacteria in the database simultaneously and automatically.

Here we present MetOrigin, a comprehensive bioinformatics pipeline, aiming to discriminate the origins of metabolites through an integrative database searching, to perform the metabolic pathway enrichment analysis (MPEA) according to their different origins, to integrate biological knowledge and statistical correlations using powerful Sankey network visualization, and finally to help researchers to generate the mechanistic hypothesis of microbe–metabolite interactions. We demonstrate MetOrigin by analyzing two gut microbiome and metabolome studies on obesity, including a pediatric study with 28 participants and the TwinsUK cohort with 786 participants. MetOrigin is a publicly available web server through http://metorigin.met-bioinformatics.cn/.

## METHODS

### Two gut microbiome and metabolome studies on obesity

#### Study I: Pediatric study on obesity

Eleven children with obesity (body mass index [BMI] *z* score ≥ 2) and 17 children with normal weight were included in this study. Metagenomics sequencing was performed on fecal samples using an Illumina Novaseq. 6000 platform. Serum samples were initially processed to extract metabolites and remove proteins as described previously [9], and then the supernatants were measured using an untargeted metabolomics approach with ultra‐performance liquid chromatography/tandem mass spectrometry (UPLC‐MS/MS) platform.

#### Study II: TwinsUK cohort study on obesity

We analyzed the fecal metabolome and microbiome of 786 individuals from the TwinsUK cohort [10]. 16S ribosomal RNA (rRNA) was extracted from fecal samples, polymerase chain reaction amplified, barcoded per sample, and sequenced with the Illumina MiSeq platform, as previously described [11]. Nontargeted metabolomics analysis was performed on fecal samples using UPLC‐MS/MS platform [12].

### Metabolite origin database

**Figure 1:**
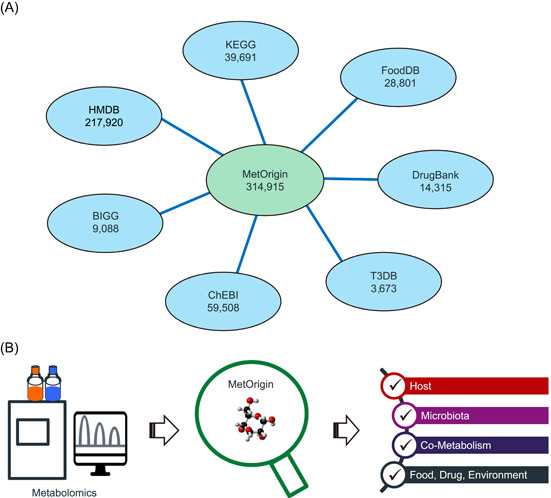
(A) Integration of seven metabolite databases in MetOrgin. (B) Illustration of applying MetOrgin for metabolite classifications. ChEBI, Chemical Entities of Biological Interest; FoodDB, Food Database; HMDB, human metabolome database; KEGG, Kyoto Encyclopedia of Genes and Genomes; T3DB, Toxin and Toxin Target Database

MetOrigin integrates seven well‐known metabolite databases that contain source information, including Kyoto Encyclopedia of Genes and Genomes (KEGG) [13], the human metabolome database (HMDB) [14], BIGG [15], and Chemical Entities of Biological Interest (ChEBI) [16], the Food Database (FoodDB) [17], Drugbank [18], and the Toxin and Toxin Target Database (T3DB) [19] (Figure 1A). First, we combined all the metabolites from seven databases. KEGG, HMDB, BIGG, and ChEBI database contain metabolites from mammals, microbiota, food, drug, toxins, pollutants, and so forth, while FoodDB, Drugbank, and T3DB contain metabolites specifically from food, drug, and toxins, correspondingly. Then, redundant metabolites were further examined and removed from the integrated database when they were cross‐validated by different databases with database IDs, chemical formulas, and compound names or synonyms. So far, there are 314,915 “nonredundant” metabolites in the MetOrigin with at least one database link. Among which, 191,031 metabolites contain specific source information that can be classified into six groups, including host (mammals), microbiota (archaea, fungi, bacteria), cometabolism (shared by both host and microbiota), food (food & plant), drug, and environment (toxins & pollutants). The detailed information of seven databases (update dates, sources of metabolites) is listed in Table S1. So that, the identified metabolites from metabolomics studies can be matched according to their KEGG IDs, HMDB IDs, and/or compound names (including all the synonyms), and then classified into different origins for the following analysis (Figure 1B).

### Origin‐based metabolic function analysis

The MPEA analysis has been routinely applied in metabolomics to identify the most relevant pathways by combining the enrichment analysis with pathway topological characteristics [20]. The algorithm for topological analysis and the pathway library are two major parts of MPEA analysis. First, the hypergeometric test is used to evaluate the pathway impact by applying R function phyper (*q *− 1,*m*,*n*,*k*) where *q* is the number of differential metabolites matched in a pathway, *m* is the total number of metabolites in a pathway, *n* is the number of metabolites in other metabolic pathways, and *k* is the total number of differential metabolites. Instead of treating all the metabolites in a pool, MetOrigin performs the MPEA of differential metabolites from different origins separately. Second, their reference metabolic pathways are defined for the host, microbial community, or shared by both, correspondingly. The reference metabolic pathways for the host are obtained directly through searching the KEGG database, for example, human and mouse, and so forth. The reference metabolic pathways of the microbial community are determined by combining all the metabolites of over 6800 microbes in the database. The reference metabolic pathways shared by the host and microbial community are the combination of metabolic pathways from both host and microbial communities.

### Integrating biological and statistical correlation in Sankey network

Metorigin explores microbiome and metabolome associations in two different ways: statistical and biological correlation. Statistically, MetOrigin provides three classical methods of correlation analysis, including Spearman, Pearson, and maximal information coefficient analysis. Biologically, each metabolite was searched against the KEGG database to identify bacterial species that could participate in a metabolic reaction.

Next, we aimed to develop an intuitive method for visualization that could meet four criteria: (1) indicate the statistically positive and negative correlations between microbes and metabolites; (2) provide all the possible bacteria that may participate in a metabolic reaction according to the database searching; (3) provide the taxonomic classifications of each bacteria; (4) count the number of bacteria belonging to a same taxonomic classification that indicates the degree of their potential involvement in a metabolic reaction. In contrast to the classical heatmap of correlation coefficients, we found that Sankey diagrams can meet all of these criteria as a good alternative to visualize microbial communities [21, 22].

Here, the Sankey network was applied to visualize the relationship between bacteria at different taxonomic levels and metabolites in a metabolic reaction. Specifically, for each metabolic reaction from a significant metabolic pathway, a Sankey network was generated linking related bacteria with the substrate, product metabolites, and their enzymes. The related enzymes are obtained by searching against the KEGG database. The enzymes for the same metabolic reaction in different species can be different, then two or more enzymes will be linked to the corresponding bacteria in a Sankey network. Meanwhile, the taxonomic annotations of those bacteria ranging from phylum, class, order, family, to species were summarized at different nodes. The widths of the bands between nodes were linearly proportional to the number of bacteria; therefore, the wider band indicated the deeper involvement of a group of bacteria.

In the Bio‐Sankey network, we explored all potential bacteria that might participate in a metabolic reaction. Each metabolite, its metabolic reaction, and related bacteria at different classification levels (i.e., at phylum, class, order, family, genus, and species) were connected using gray bands as background. For studies with both metabolite and microbiota datasets available, statistically significant correlations would be analyzed and highlighted in red and green color, indicating positive and negative correlations. The Bio‐Sankey network aims to explore the biological correlations between bacteria and a metabolite of interest (and its metabolic reaction), which could be simultaneously validated by the statistical correlations using real datasets.

In the STA‐Sankey network, we explored all the bacteria that were statistically associated with a metabolic reaction, and they were also highlighted in red or green color if there exists a biological relationship indicated by database searching. Compared to the Bio‐Sankey network, STA‐Sankey focuses on the statistical correlations between bacteria and metabolites in real datasets, which may indicate the potential novel relationship between them.

### Implementation

MetOrigin is a cloud server developed by R shiny application. All the statistical analyses and visualization were implemented in R (Version 3.6.0), by applying packages KEGGREST, Hmisc, Vennerable, ggplot2, and Plotly. The R functions and packages are summarized in Table S2. The entire system is deployed on a cloud server with 16 GB of RAM and four virtual CPUs with 2.6 GHz each. A user tutorial describing the main features, file formats, and data interpretation is provided on the home page. The web interface is freely accessed through a web browser http://metorigin.met-bioinformatics.cn/.

## RESULTS

### MetOrigin workflow

MetOrigin implements an interactive interface allowing users to analyze and visualize the microbiome and metabolome datasets (Figure S1). Details are available in the online tutorial. Briefly, MetOrigin provides two types of data analysis modes according to the availability of metabolite and microbiota datasets, as well as the purpose of research. The first one is Simple MetOrigin Analysis (SMOA) mode and it requires a list of metabolites with KEGG or HMDB IDs. SMOA provides origin analysis to identify the origins of metabolites, the metabolic function analysis, and Sankey network visualization to explore the biologically related microbiota and metabolites. The second option is Deep MetOrigin Analysis (DMOA) and it takes three individual files as input datasets, including a “metabolite” table with compound names, KEGG or HMDB IDs, and abundance/concentration values, a “microbiome” table with their annotations and abundance values from either 16S rRNA gene sequencing or shotgun metagenomic sequencing, and a “Sample Info” table with sample names and grouping information, for example, control versus diseased. Besides origin analysis, function analysis, and Sankey network visualization, DMOA also provides correlation analysis and network summary to explore the statistically and biologically correlated microbiota and metabolites.

Before proceeding to perform statistical analysis, data pretreatment is an important step in both metabolomics and metagenomics studies, including missing value imputation and data standardization. Our previous work has evaluated and compared the features of a variety of missing value processing methods, for example, replacing missing values with minimum values directly, or applying random forest, K‐nearest neighbors, or quantile regression imputation of left‐censored data for imputation [23]. Here, we provide these methods in the MetOrigin, and users need carefully select an appropriate method and evaluate the effects on statistical analysis subsequently. Meanwhile, the log transformation and percentage calculation are commonly used and applied for data normalization in MetOrigin. After completing all the analysis in the MetOrigin, the data analysis results including tables and high‐resolution figures can be downloaded for further data analysis and interpretation.

### MetOrigin database

**Figure 2:**
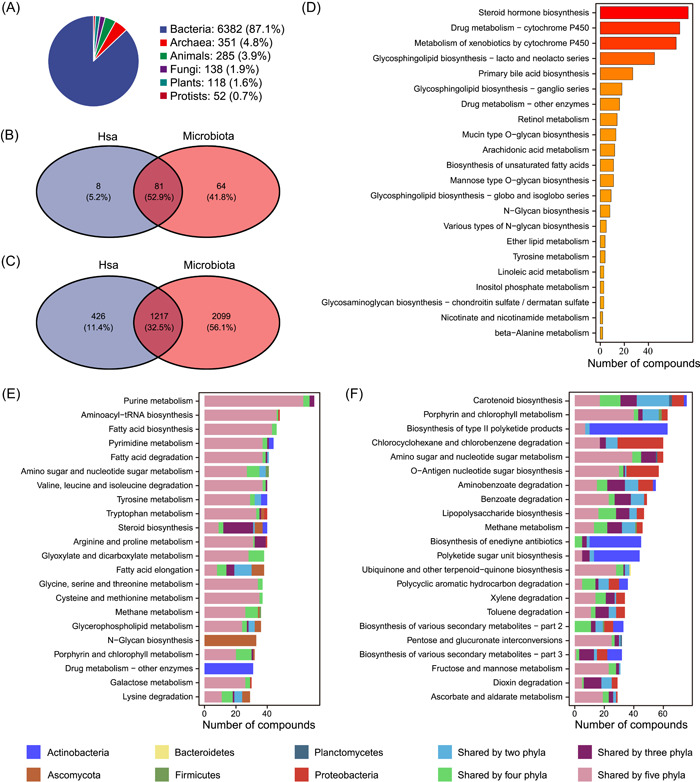
MetOrigin database summary. (A) Summary of different organisms in the KEGG database. (B) Venn diagram of the number of metabolic pathways in the human and microbial community. (C) Venn diagram of the number of metabolites in the human and bacterial communities. (D–F) Bar plots of enriched metabolic pathways in (D) human, (F) microbiota, and (E) shared by both. KEGG, Kyoto Encyclopedia of Genes and Genomes

A total of 314,915 “nonredundant” metabolites were collected and organized in the MetOrigin from seven well‐known metabolite databases (Figure 1A, see details in the Methods). Among which, 191,031 metabolites contain specific source information, so that the identified metabolites can be classified into different categories, that is, host (mammals), microbiota (archaea, fungi, bacteria), cometabolism (shared by both host and microbiota), food (food & plant), drug, and environment (toxins & pollutants; Figure 1B). A total of 7326 different organisms were included mainly according to the KEGG database, among which there were 6382 bacterial organisms and 285 animals (Figure 2A). We calculated the total number of metabolites and metabolic pathways belonging to humans (hsa) and compared them against bacterial communities (Figure 2B,C). There were 426 metabolites in 8 metabolic pathways specific to humans, and the top five enriched metabolic pathways were steroid hormone biosynthesis, drug metabolism, metabolism of xenobiotics, glycosphingolipid biosynthesis, and primary bile acid biosynthesis (Figure 2D). There were 2099 metabolites specific to the bacterial community in 64 metabolic pathways, and the top five enriched metabolic pathways were carotenoid biosynthesis, porphyrin and chlorophyll metabolism, biosynthesis of type II polyketide products, amino sugar and nucleotide sugar metabolism, and O‐antigen nucleotide sugar biosynthesis (Figure 2F). Meanwhile, there were 1217 metabolites shared by human and microbial communities in 81 different metabolic pathways, and the top five enriched metabolic pathways were purine metabolism, aminoacyl‐transfer RNA biosynthesis, fatty acid biosynthesis, pyrimidine metabolism, and fatty acid degradation (Figure 2E). Compared to bacteria‐specific metabolites, metabolites from the human and bacteria cometabolism seemed to be shared by more phyla.

### Gut microbiome and metabolome analysis on pediatric obesity

#### Metabolite origin analysis and origin‐based metabolic function analysis

**Figure 3:**
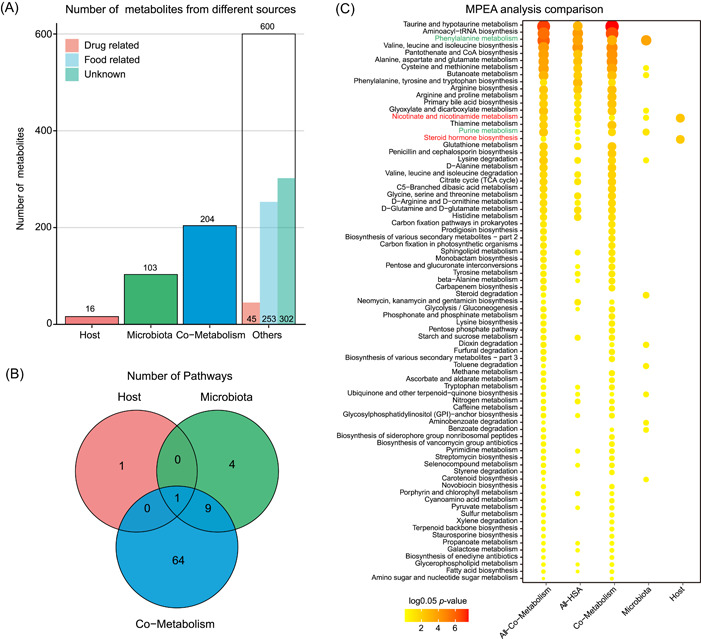
Origin analysis and origin‐based function analysis of pediatric study on obesity. (A) Bar plot of the number of metabolites in different categories. (B) Venn diagram of the number of enriched metabolic pathways from origin‐based MPEA analysis. (C) Comparison of MPEA analysis using different methods, including applying subgroup of metabolites from the host, microbiota, or cometabolism and matching them to their corresponding pathways, or matching all the metabolites to the metabolic pathways from the host (All‐Host) or cometabolism (All‐Co‐Metabolism) in a traditional way. MPEA, metabolic pathway enrichment analysis

A total of 923 identified metabolites were initially classified into four groups: 16 host (human)‐specific metabolites, 103 bacterial metabolites, 204 bacteria–host cometabolites, and 600 others (drug and food) (Figure 3A). There were 249 differential metabolites associated with obesity, including 6 host‐specific metabolites, 32 bacterial metabolites, 65 bacteria–host cometabolites, and 146 others. The differential metabolites from the host, microbiota, and shared by both were used for MPEA analysis against their corresponding reference metabolic pathways. For comparison, we also performed MPEA by using all the differential metabolites together against the human reference metabolic pathway database alone (ALL‐HSA), or against the human and bacteria integrated database as reference (ALL‐Co‐Metabolism; Figure 3C). As a result, we observed that performing the MPEA against the host metabolic pathway database alone (HSA) would miss the contribution of metabolites from bacteria. However, integrating human and bacterial‐associated pathways (MPEA‐ALL) may simply produce overlapping effects from differential origins. In contrast, origin‐based MPEA would calculate the significance of metabolic pathways according to the metabolites from different origins. For example, there were 2, 14, and 74 related metabolic pathways were matched against the host, bacteria, and cometabolism pathway database (Figure 3B). Among which, 2, 2, and 31 metabolic pathways were identified significantly associated with obesity, correspondingly (*p* < 0.05). Based on origin‐based function analysis, the nicotinate and nicotinamide metabolism, and steroid hormone biosynthesis were specific to the host alone. The phenylalanine metabolism and purine metabolism were specific to the microbial community, and 31 metabolic pathways associated with amino acids, lipids, and sugars were shared by both host and microbiota.

#### Visualization of biological and statistical correlation between bacteria and metabolites using Sankey network

**Figure 4:**
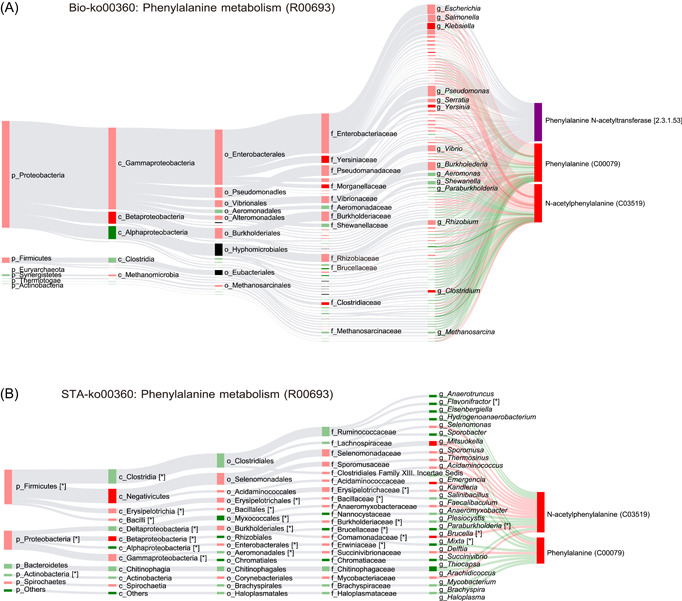
(A) The BIO‐Sankey Network for R00693 metabolic reaction in phenylalanine metabolism. (B) The STA‐Sankey Network for R00693 metabolic reaction in phenylalanine metabolism. Asterisks (*) indicate statistically significant correlations with metabolites. The red/green color of nodes indicates up/downregulation. The red/green bands indicate the positive/negative correlations with metabolites. The dark red/green color indicates the statistically significance *p* < 0.05

Spearman correlation analysis was performed between gut microbiota and metabolites of fecal samples. There were 902, 1364, 2431, 4113, 13,285, and 62,976 closely associated bacteria–metabolite pairs at phylum, class, order, family, genus, and species level, respectively (*p* < 0.05). According to the metabolites from microbiota, we identified two significant metabolic pathways that were associated with obesity, that is, phenylalanine metabolism and purine metabolism. Take the phenylalanine metabolism, for example, four differential metabolites (i.e., benzoate, phenyllactate, *N*‐acetylphenylalanine, and 3‐hydroxyphenylpropanoate) were involved in this pathway, which participated in a total of five different metabolic reactions (R00693, R01370, R01371, R01424, and R06786). For each metabolic reaction, MetOrigin explored the biological and statistical correlations between microbiota and metabolites using Bio‐Sankey and STA‐Sankey networks. In the Bio‐Sankey network, Proteobacteria and Firmicute were identified as major phyla closely associated with the metabolic reaction R00693 (Figure 4A). The phenylalaine produces *N*‐acetyl‐phenylalanine via acetyl‐CoA: l‐phenylalanine *N*‐acetyltransferase. In this study, we identified that the phenylalanine and *N*‐acetyl‐phenylalanine were significantly upregulated in obesity. *Klebsiella, Yersinia, and Clostridium* were the most significantly increased genus in patients with obesity (dark red if *p* < 0.05), which were associated with R00693 in parallel. In the STA‐Sankey network, integrative statistical correlation analysis confirmed that Proteobacteria and Firmicute were closely associated with phenylalanine and *N*‐acetyl‐phenylalanine in the metabolic reaction R00693, which were complementary to the results from BIO‐Sankey (Figure 4B). To summarize, the Bio‐Sankey and STA‐Sankey networks could provide the biological and statistical correlations between microbiome and metabolome straightforwardly.

#### Network summary

**Figure 5:**
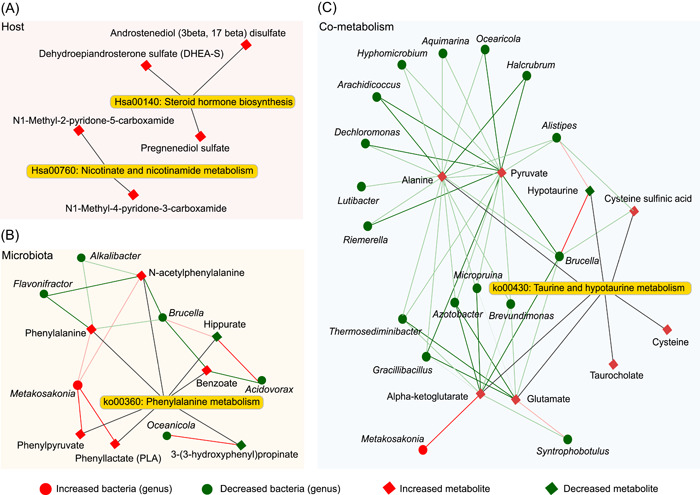
Network summary of pediatric study on obesity for human (A), microbiota, (B) and shared by human and microbiota (C). Diamond and dot shapes indicate corelate metabolites and microbes, correspondingly. The red/green color of nodes indicates up/downregulation. The red/green lines indicate the positive/negative correlations between microbes and metabolites

Finally, we summarized and integrated the differential metabolites from the host, microbiota, and cometabolism origins, and their related bacteria within a network to obtain a whole picture of microbiome and metabolome cross‐talk associated with obesity. The “host” metabolic network indicated that five significantly upregulated metabolites in the nicotinate and nicotinamide metabolism and steroid hormone biosynthesis were solely associated with the host (Figure 5A). The “microbiota” network of phenylalanine metabolism illustrated that seven metabolites were closely associated with six genera (*p* < 0.01) that had been validated by both biological and statistical correlation analysis (Figure 5B). The cometabolism network of taurine and hypotaurine metabolism showed that eight metabolites were associated with 17 differential bacteria (*p* < 0.01), and most of them were negatively correlated (Figure 5C). In summary, the host‐specific metabolic network and microbe–metabolite association network provided more specific information on metabolic changes associated with obesity.

### Gut microbiome and metabolome analysis on TwinsUK cohort

**Figure 6:**
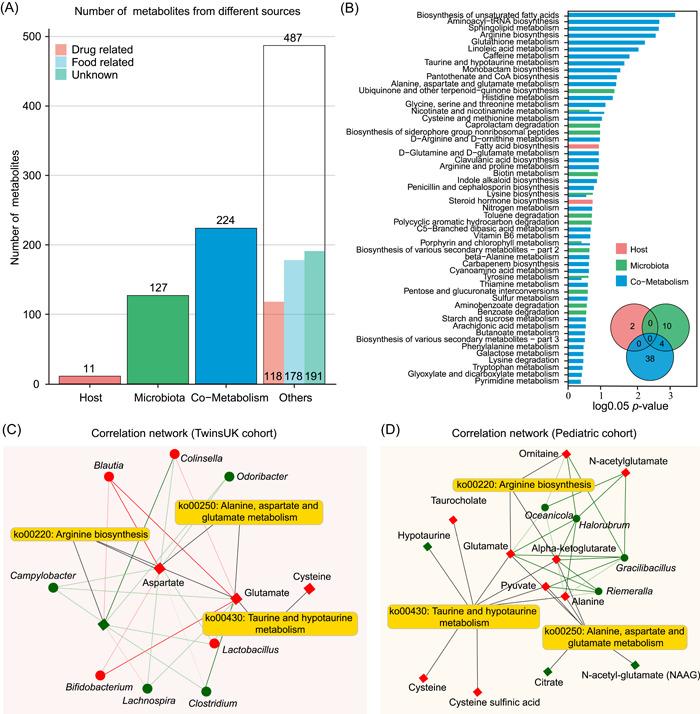
MetOrigin analysis on TwinsUK cohort study. (A) Bar plot of the number of metabolites in different categories. (B) Venn diagram of the number of enriched metabolic pathways from origin‐based MPEA analysis. (C) Correlation network of microbes and metabolites from three selected metabolic pathways using TwinsUK cohort study. (D) Correlation network of microbes and metabolites from three selected metabolic pathways using Pediatric cohort study. MPEA, metabolic pathway enrichment analysis

Next, we applied MetOrgin to analyze gut microbiome and metabolome on obesity using TwinsUK datasets. A total of 923 identified metabolites from the TwinsUK study were initially classified into four groups: 11 host‐specific metabolites, 127 bacterial metabolites, 224 bacteria–host cometabolites, and 487 others (Figure 6A). There were 93 differential metabolites associated with obesity (BMI < 24 vs. BMI > 28), including three host‐specific metabolites, 19 bacterial metabolites, 23 bacteria–host cometabolites, and 48 others. The origin‐based MPEA analysis identified 2, 14, and 42 metabolic pathways from the host, microbiota, and shared by both (Figure 6B). Similar to Pediatric Obesity Study, the steroid hormone biosynthesis was specific to the host. Among 42 metabolic pathways of cometabolism, we identified three amino acid‐associated metabolic pathways (i.e., arginine biosynthesis, taurine and hypotaurine metabolism, alanine, aspartate, and glutamate metabolism) were differentially expressed in both pediatric and adult obesity studies. Their microbe–metabolite correlations were further visualized using a network (Figure 6C,D).

## DISCUSSION

Although gut microbiome and metabolome research on human health and diseases has increased rapidly in recent years, the understanding of the cross‐links between microbiome and metabolome is limited. Current strategies to explore their complex relationships are: to recognize the microbial metabolites (e.g., short‐chain fatty acids, branched‐chain fatty acids, and bile acids) in metabolomics studies indicating the potential involvement of microbiome [2], to predict the metabolic function of the microbial community in metagenomics studies [24], and to perform statistical correlation analysis when both metabolomics and metagenomics studies are conducted [25, 26]. However, it is an extremely time‐consuming process to identify the critical microbial species that participate in the metabolic reactions for metabolites of interest. MetOrigin aims to speed up this process by discriminating the origins of metabolites initially through integrative database searching, performing origin‐based metabolic function analysis for better understanding the contribution of different organisms towards metabolism, and integrating both statistical and biological correlation to improve the accuracy of biomarker discovery.

The first step of MetOrigin is to identify metabolites that may come from different sources. The determination of the origins of metabolites currently depends on the integration of seven powerful databases, including HMDB, BIGG, ChEBI, FoodDB, Drugbank, and T3DB. So far, MetOginin has included a total of 191,031 metabolites that contain specific sources, including host (mammals), microbiota (archaea, fungi, bacteria), cometabolism (shared by both host and microbiota), or from food (food & plant), drug, and environment (toxins & pollutants). However, there is rich information that exists in other databases and tons of literature, it is of great importance in the future to explore and integrate them to empower our existing knowledge database so that we have more complete and precise classifications of metabolites for further scientific research and discovery.

Sankey network is the core of the MetOrigin pipeline aiming to illustrate the biological and statistical relationship between metabolites of interest and related bacteria. For the Bio‐Sankey network of a metabolic reaction, the background lists all the relevant bacteria that have the metabolic reaction in the database, that is, either substrate or product compounds can be searched against the integrated database. The widths of network bands determined by their microbial taxonomy classifications can indicate the degree of cross‐talk between bacteria and metabolites. Meanwhile, statistically, significant correlations are further highlighted to emphasize the most reliable relationship. In comparison, the STA‐Sankey network of a metabolic reaction applies the highly correlated microbes and metabolites as background that may provide novel productive/consumptive relationships outside of the existing knowledge system, since high‐throughput deep sequencing analysis have been increasingly applied in this field. The combination of Bio‐Sankey and STA‐Sankey can help us to speed up the process of potential biomarker discovery and generate novel hypotheses for further scientific research.

To be mentioned, the MetOrigin pipeline aims to explore the gut microbiome and metabolome crosstalk efficiently and identify the microbe‐metabolite correlations accurately. However, whether specific bacteria are truly involved in a metabolic reaction of interest, or a metabolite is utilized or produced by bacteria requires further biological experiments to confirm their potential cause–consequence relationships, such as germ‐free animal studies and in vitro microbial culture models. Thus, it is more accurate to define “microbiota‐related” metabolites before validation. Finally, the MetOrigin is not limited to research on gut microbiome alone, it is valuable to explore correlations between microbiome from others (e.g., oral, skin, environment, etc.) and metabolites.

## CONCLUSIONS

To summarize, MetOrigin is a publicly available and interactive cloud server, designed for research scientists to streamline the whole process of data mining, and does not require computation or bioinformatics research background. MetOrigin meets the urgent need to explore the complex relationship between microbiome and metabolome. It is novel to perform MPEA on metabolites from different origins, and link biological and statistically correlated microbiota using the Sankey network. This powerful bioinformatics tool provides valuable information on microbial participation towards metabolism and will help researchers to discover novel dietary or pharmacological intervention strategies to prevent microbial dysbiosis and maintain metabolic balance in the future.

## CONFLICTS OF INTEREST

The authors declare that there are no conflicts of interest.

## AUTHOR CONTRIBUTIONS

Yan Ni conceived the idea and supervised this study. Gang Yu and Cuifang Xu performed this project including data processing, data analysis, and software development. Yan Ni wrote the manuscript. Danni Zhang and Cuifang Xu collected and integrated metabolite databases. Feng Ju provided valuable suggestions on metagenomics data analysis and modified the manuscript. All authors reviewed and approved the manuscript.

## Supporting information

Supporting information.

Supporting information.

Supporting information.

## Data Availability

The demo data used in this paper can be viewed and downloaded from http://metorigin.met-bioinformatics.cn/. Supporting Information (figures, tables, scripts, graphical abstract, slides, videos, Chinese translated version, and updated materials) may be found in the online DOI or iMeta Science http://www.imeta.science/.
