# Correction to “MetOrigin: Discriminating the origins of microbial metabolites for integrative analysis of the gut microbiome and metabolome”

**DOI:** 10.1002/imt2.149

**Published:** 2023-11-06

In Yu et al. [1], the following ethics statement should have been added.

The ethics application (No. 2021‐IRB‐038) was approved by the Medical Ethics Committee of Children's Hospital Affiliated to Zhejiang University School of Medicine, which was also registered in the Chinese Clinical Trial Registry (ChiCTR2100045616).

And data should have been uploaded to GSA (https://ngdc.cncb.ac.cn/gsa/).

All the sequencing data have been deposited in GSA under accession number CRA013297 (metagenome sequencing), BioProject accession number PRJCA020992.

The authors wish to apologize for the oversight.
