# Supplementary material for: MetOrigin: Discriminating the origins of microbial metabolites for integrative analysis of the gut microbiome and metabolome
Source: Imeta. 2022 Mar 21;1(1):e10. doi: 10.1002/imt2.10 (PMC10989983; doi:10.1002/imt2.10)

**Supporting Information**

**MetOrigin: discriminating the origins of microbial metabolites for integrative analysis of the gut microbiome and metabolome**

**Supplementary Figure S1**. The flowchart of MetOrigin analysis.
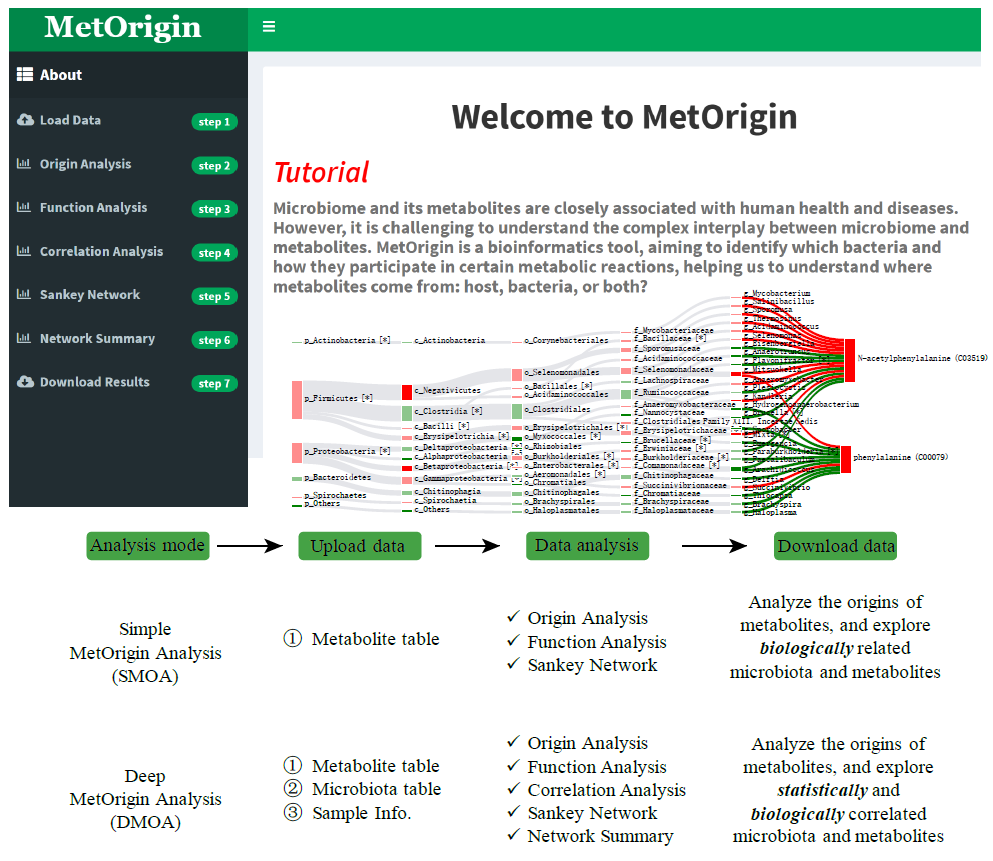

Supplement: Supplementary file 1 — Supporting information. [file IMT2-1-e10-s001.docx]
