# Supplementary material for: MetOrigin: Discriminating the origins of microbial metabolites for integrative analysis of the gut microbiome and metabolome
Source: Imeta. 2022 Mar 21;1(1):e10. doi: 10.1002/imt2.10 (PMC10989983; doi:10.1002/imt2.10)
Supplement: Supplementary file 3 — Supporting information. [file IMT2-1-e10-s003.docx]

**Supporting Information**

Additional supporting information may be found in the online version of the article at the publisher's website.
